# An ionic silver coating prevents implant-associated infection by anaerobic bacteria in vitro and in vivo in mice

**DOI:** 10.1038/s41598-022-23322-6

**Published:** 2022-11-01

**Authors:** Tomoya Soma, Ryotaro Iwasaki, Yuiko Sato, Tami Kobayashi, Eri Ito, Tatsuaki Matsumoto, Atsushi Kimura, Fuka Homma, Keitarou Saiki, Yukihiro Takahashi, Kana Miyamoto, Morio Matsumoto, Masaya Nakamura, Mayu Morita, Ken Ishii, Seiji Asoda, Hiromasa Kawana, Zhu Xingyu, Mamoru Aizawa, Taneaki Nakagawa, Takeshi Miyamoto

**Affiliations:** 1 Division of Oral and Maxillofacial Surgery, Department of Dentistry and Oral Surgery, Keio University School of Medicine, 35 Shinano-Machi, Shinjuku-Ku, Tokyo, 160-8582 Japan; 2 Department of Orthopedic Surgery, Keio University School of Medicine, 35 Shinano-Machi, Shinjuku-Ku, Tokyo, 160-8582 Japan; 3 Department of Advanced Therapy for Musculoskeletal Disorders II, Keio University School of Medicine, 35 Shinano-Machi, Shinjuku-Ku, Tokyo, 160-8582 Japan; 4 Department of Musculoskeletal Reconstruction and Regeneration Surgery, Keio University School of Medicine, 35 Shinano-Machi, Shinjuku-Ku, Tokyo, 160-8582 Japan; 5 Institute for Integrated Sports Medicine, Keio University School of Medicine, 35 Shinano-Machi, Shinjuku-Ku, Tokyo, 160-8582 Japan; 6 Department of Microbiology, The Nippon Dental University School of Life Dentistry at Tokyo, 1-9-20 Fujimi, Chiyoda-Ku, Tokyo, 102-8159 Japan; 7 Department of Orthopedic Surgery, Kumamoto University, 1-1- Honjo, Chuo-Ku, Kumamoto, 860-8556 Japan; 8 Department of Orthopaedic Surgery, School of Medicine, International University of Health and Welfare (IUHW), 852 Hatakeda, Narita City, Chiba 286-8520 Japan; 9 Department of Oral and Maxillofacial Implantology, School of Dentistry, Kanagawa Dental University, 82 Inaoka-Cho, Yokosuka, Kanagawa 238-8580 Japan; 10 Department of Applied Chemistry, School of Science and Technology, Meiji University, 1-1-1 Higashimita, Tama-Ku, Kawasaki, Kanagawa 214-8571 Japan

**Keywords:** Experimental models of disease, Osteoporosis

## Abstract

Currently, implants are utilized clinically for bone transplant procedures. However, if infectious osteomyelitis occurs at implant sites, removal of bacteria can be challenging. Moreover, altered blood flow at peri-implant infectious sites can create an anaerobic environment, making it more difficult to treat infection with antibiotics. Thus, it would be beneficial if implants could be modified to exhibit antibacterial activity, even in anaerobic conditions. Here, we show antibacterial activity of silver ions coated on titanium rods, even against the anaerobic bacteria *Porphyromonas gingivalis* (*P. gingivalis*), both in vitro and in vivo. Specifically, we implanted silver-coated or control uncoated titanium rods along with *P. gingivalis* in mouse femoral bone BM cavities and observed significantly inhibited *P. gingivalis* infection with silver-coated compared with non-coated rods, based on in vivo bio-imaging. Osteonecrosis by infectious osteomyelitis and elevation of the inflammatory factors C-reactive protein and IL-6 promoted by *P. gingivalis* s were also significantly reduced in the presence of silver-coated rods. Overall, our study indicates that silver ion coating of an implant represents a therapeutic option to prevent associated infection, even in anaerobic conditions or against anaerobic bacteria.

## Introduction

To date, dental implants have been developed to replenish, restore or reinforce defective bone due to conditions like periodontitis, tumors or trauma. In orthopedic practice, other devices have been developed for joint replacement and fracture repair. Such implants are now in fact indispensable clinical tools to maintain or improve patients’ activities of daily living (ADL) and maintain their quality of life (QOL). However, once implant-associated infections occur, they can be difficult to cure, as implants have no inherent anti-bacterial activity. Also, some bacteria form antibiotic-resistant biofilms on implant surfaces, or the infectious region can become hypo-vascular due to granuloma formation^1,2^. In severe cases, implants must be removed to cleanse them of implant-associated infection, conditions that severely worsen patients’ ADL and QOL^3–5^. Risk of implant-associated infection is particularly high with dental implants, which are exposed to high levels of bacteria in the oral environment, including those that cause periodontitis^6,7^. Also, in orthopedics, infectious osteomyelitis is difficult to treat whether a patient has had an implant or not. Antibiotics and protocols for their use in these conditions have been developed to treat implant-associated infections^8–10^, but their effects vary in different contexts, such as loss of tissue-migrating capacity of agents owing to granulation or biofilm formation on the device surface^11,12^.

Development of implants with inherent antimicrobial activity is one approach to these issues but requires several considerations: implant strength cannot be reduced by acquisition of antimicrobial activity nor can the material that promotes antimicrobial activity exhibit cytotoxicity in vivo. Various means to satisfy these criteria have been developed^13^, including coating implants with silver ions, as they reportedly exhibit antimicrobial activity against *Staphylococcus aureus* and antagonize biofilm formation without cytotoxicity^14,15^. Silver ions reportedly provide antibacterial effects by promoting generation of reactive oxygen species in the presence of oxygen^16,17^, although underlying mechanisms remain unknown. Nonetheless, anaerobic bacteria often underlie implant infection, among them *Porphyromonas gingivalis* (*P. gingivalis*), which causes periodontitis and oral implant-associated infections, leading to implant failure^18–20^. Thus far it is not known whether silver ions exert antibacterial effects in anaerobic conditions or against anaerobic bacteria.

Here, we evaluated antimicrobial effects of silver ions against *P. gingivalis* in anaerobic conditions in vitro and found them effective, even in anaerobic conditions*.* Given that titanium is frequently used for implant material, we then coated titanium rods with silver ions and found that they exhibited antimicrobial activity greater than that of uncoated controls against PGs in anaerobic conditions in vitro and in in vivo. Specifically, *P. gingivalis* levels in mouse femoral bones, as detected using a fluorescence-based probe, decreased more rapidly in the presence of titanium rods coated with silver ions than with uncoated control rods. Histologically, osteonecrosis development owing to *P. gingivalis* -dependent infectious osteomyelitis was significantly inhibited when titanium rods were coated with silver ions. Similarly, serum levels of inflammatory C-reactive protein (CRP) and IL-6 increased following *P. gingivalis* transplantation in mouse femur, an increase significantly blocked when silver ion-coated titanium rods were present. Taken together, these results indicate that ionic silver coating is a clinically useful option to prevent implant-associated infections in anaerobic conditions or following infection with anaerobic bacteria.

## Results

### Silver ions exhibit antimicrobial activity against the anaerobic bacteria *Porphyromonas gingivalis *in vitro

**Figure 1:**
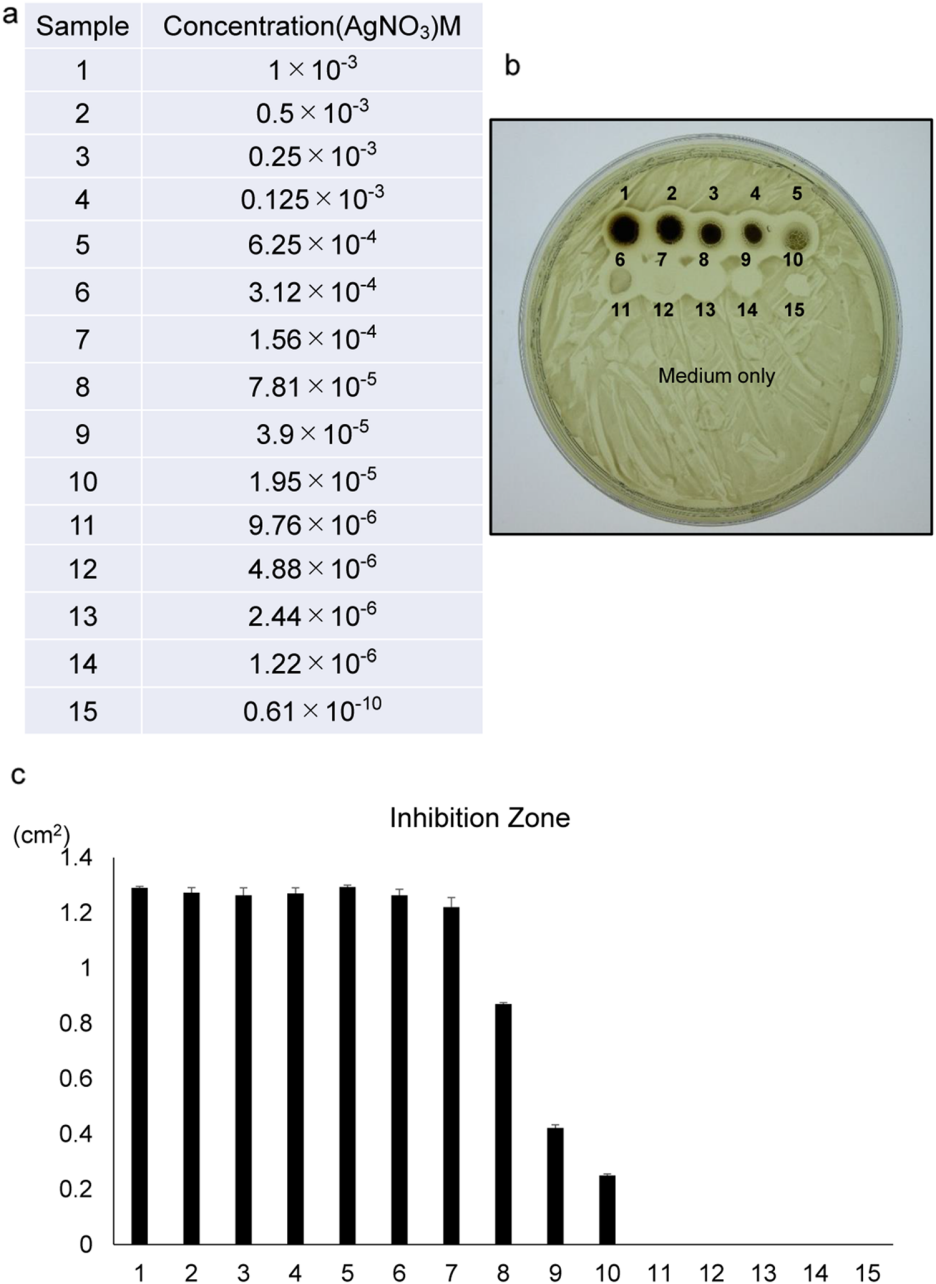
Silver nitrate inhibits *Porphyromonas gingivalis* growth. (**a**) Dilution series of a silver nitrate AgNO_3_ (169.87) solution. (**b**) *Porphyromonas gingivalis* (*P. gingivalis*) was streaked uniformly on a BHIHM agar plate. Then, two-fold serial dilutions of AgNO_3_ (2 μl each) were spotted on the agar and incubated 48 h anaerobically at 37 °C. The *P. gingivalis* minimum inhibitory concentration (MIC) was 1.95 × 10^−5^ M. Shown are dose-responsive inhibitory effects of silver nitrate solution on *P. gingivalis* growth in vitro. (**c**) Shown is the mean inhibitory zone ± SD (cm^2^). Representative data are shown of at least two independent and identical experiments.

To test if silver ions (Ag^+^) have antimicrobial activity against anaerobic bacteria, we treated *Porphyromonas gingivalis (P. gingivalis)-*inoculated agar gels with various concentrations of silver nitrate (AgNO_3_; 169.87) solution in anaerobic conditions (5% CO_2_, 10% H_2_, balanced N_2_). After 48 h of cultivation, we assessed antimicrobial activity on plates based on formation of inhibition circles and detected dose-dependent antimicrobial activity of silver nitrate against *P. gingivalis’s* (Fig. 1). AgNO_3_ antimicrobial activity reached a plateau at 1.56 × 10^–4^ M.

**Figure 2:**
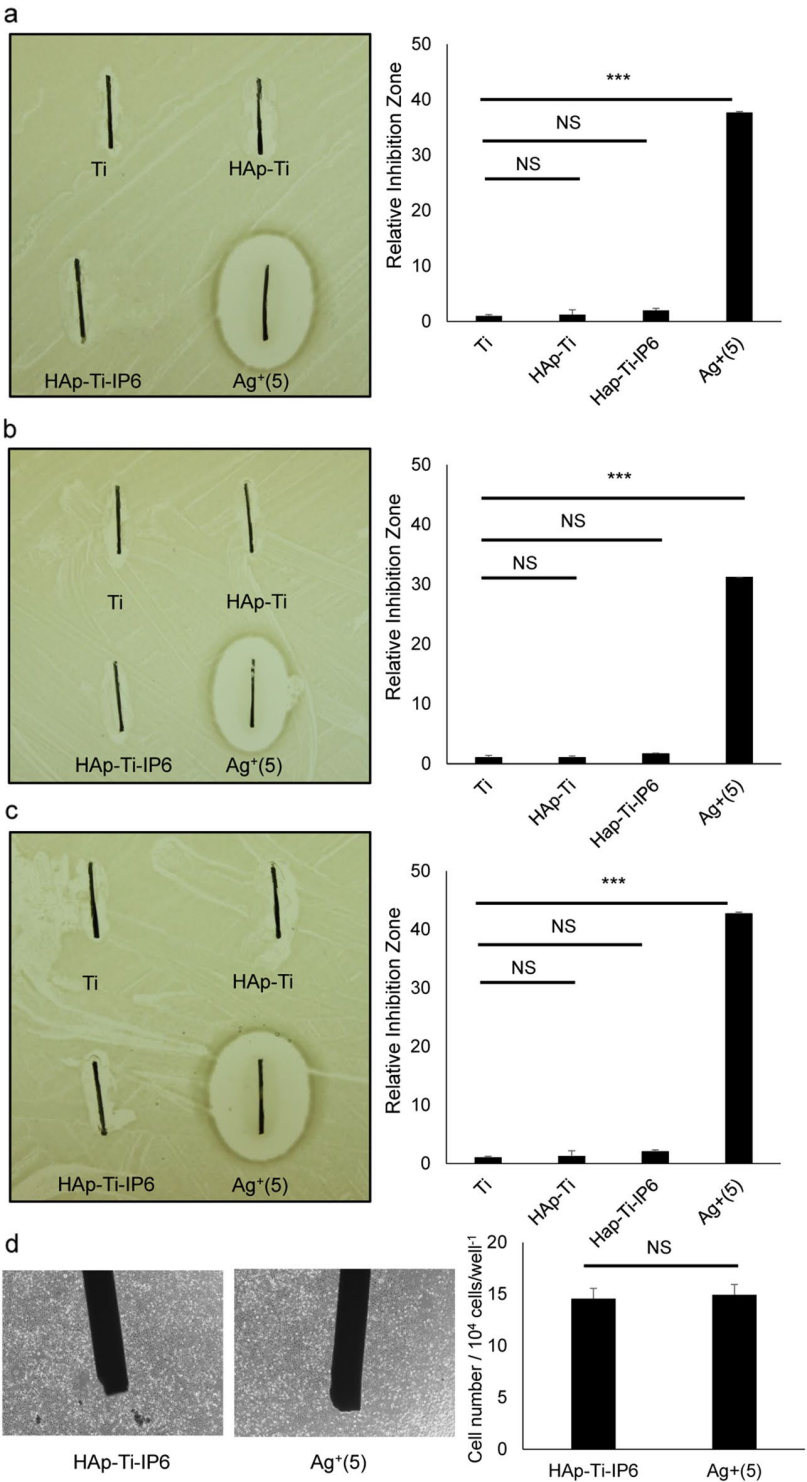
In vitro antibacterial effect of IP6-immobilized silver ions on a HAp rod. (**a** and **b**) *Porphyromonas gingivalis* (*P. gingivalis*) was cultured on a BHIHM agar plate, and then four different Ti pins (all 0.5 mm wide × 8 mm long)—(1) without surface modification (Ti), (2) coated with HAp alone (HAp-Ti), (3) coated with HAp-IP6 (HAp-Ti-IP6), or (4) coated with HAp-IP6-Ag + (Ag + (5)). Coating of the titanium rod surface was performed at 7 days (**a**), 8 months (**b**) or 4 years (**c**) prior to experiments. Bacterial growth inhibition is indicated by a circle around the pin. Shown is the mean inhibitory zone relative to Ti ± SD. Quantification of growth inhibition zones indicates significant bacterial growth inhibition by HAp-IP6-Ag^+^(5)-Ti pins relative to all controls. (n = 3, ***p < 0.001; NS, not significant; by the Mann–Whitney test). (**d**) Osteoblastic MC3T3-E1 cells were cultured with HAp-IP6-Ag + (5)-coated or uncoated Ti-HAp-IP6 implants, and after three days of culture, observed under a phase-contrast microscope. Representative data of at least two independent experiments are shown.

Titanium implants are frequently used for oral and orthopedic surgery as they are rustproof and safe for patients undergoing magnetic resonance imaging (MRI)^21–23^. To evaluate antimicrobial activity of Ag^+^ against *P. gingivalis’s *in vitro, we first coated titanium rods (Ti) with Ag^+^. To do so, we precoated rods with hydroxyapatite (HAp) to form a HAp film surface on the implant, which was then modified by treatment with IP6 (C_6_H_6_ (OPO_3_H_2_)_6_). Ag^+^ ions were then immobilized on the modified film by IP6 chelation to yield HAp-IP6-Ag^+^–Ti. We then placed HAp-IP6-Ag^+^–Ti or one of three different control rods—non-coated (Ti), coated with HAp alone, or coated with HAp and IP6—on gels inoculated with PG and compared antimicrobial activity against *P. gingivalis* growing in anaerobic conditions. Only the HAp-IP6-Ag^+^–Ti rod exerted anti-*P. gingivalis* activity, based on formation of an inhibition circle on the agar gel around that rod (Fig. 2a,b). Antimicrobial activities of HAp-IP6-Ag^+^–Ti rods were tested at day seven, month eight and four years after application of the silver ion coating (Fig. 2a–c), and that activity was equivalent among rods. Thus, antimicrobial activities of HAp-IP6-Ag^+^–Ti rods were maintained for at least four years. However, HAp-IP6-Ag^+^–Ti rods did not promote cytotoxicity in in vitro cell culture, and the number of living cells cultured with HAp-IP6-Ag^+^–Ti (Ag + (5)) or control (HAp-IP6) rods was equivalent. (Fig. 2d). These analyses indicate that Ag^+^ ions exert anti-*P. gingivalis* activity in anaerobic conditions, even when coated onto titanium rods, without promoting cytotoxicity.

### Silver ion-coating of titanium rods prevents *Porphyromonas gingivalis* infection in vivo

**Figure 3:**
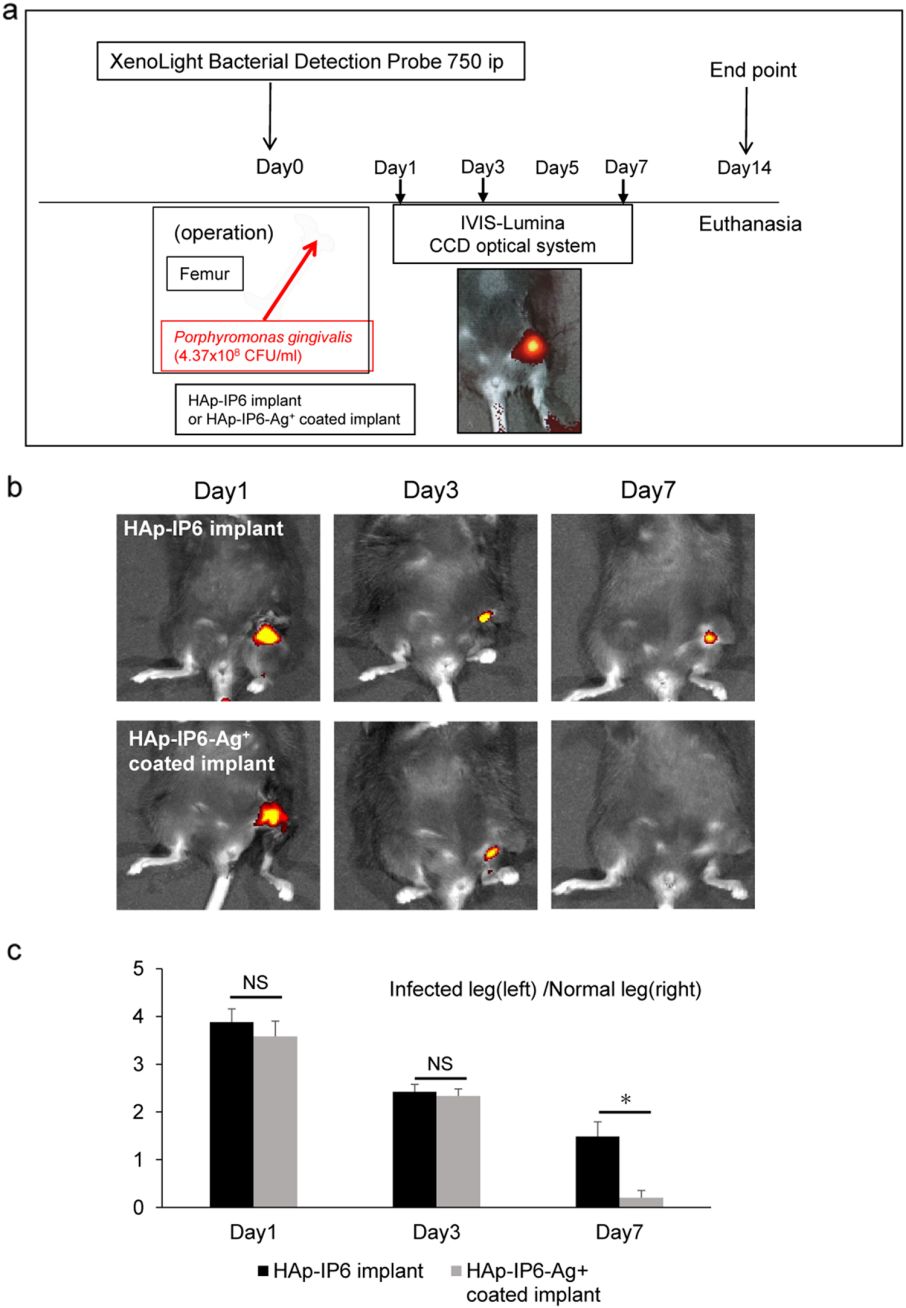
Establishment of a mouse osteomyelitis model. (**a** and **b**) Experimental protocol. 4.37 × 10^8^ CFU/ml *Porphyromonas gingivalis* (*P. gingivalis*) were transplanted into femoral bone marrow cavities with titanium rods in eight-week old wild-type mice. Specifically, a skin incision was made over the left knee, and the distal femur exposed. A drill and 23G needle were used to make a hole at the distal end of the femur, and a 0.5 × 8-mm titanium alloy bar was inserted into the mouse femur along with an inoculation of *P. gingivalis*. Simultaneously, mice were intraperitoneally injected with a NIR-fluorescent bacterial detection probe. (**c**) Shown are NIR-fluorescence signals at infected areas as detected by a bacterial probe on indicated days in indicated mice. HAp-IP6-Ag^+^ group, N = 6; control Ti-HAp-IP6 group, N = 6.

To determine whether Ag^+^ coating of titanium rods antagonizes implant-associated *P. gingivalis* infection, we established a *P. gingivalis*-based infectious osteomyelitis model based in mice (Fig. 3a,b). To do so, we transplanted *P. gingivalis’s* into femoral bone marrow cavities in eight-week-old female wild-type mice. We also implanted Ag^+^ coated HAp-IP6-Ag^+^–Ti or control HAp-IP6-Ti rods in those same bone marrow cavities and, at the same time, injected mice intraperitoneally with IVISense Bacterial 750 Fluorescent Probe. One day later, we began performing in vivo imaging of fluorescent signals over 7 days using in vivo imaging (Fig. 3c). No fluorescence activity was detected in sterile controls (Ti rods without *P. gingivalis* transplantation) at day one after surgery (Figure S1). In mice implanted with HAp-IP6-Ag^+^–Ti rods, fluorescent signals indicative of bacteria decreased faster than in control mice, and by day 7 fluorescence was not detectable in mice implanted with HAp-IP6-Ag^+^–Ti rods. By contrast, on day 7 HAp-IP6-Ti mice still showed detectable fluorescence (Fig. 3c).

**Figure 4:**
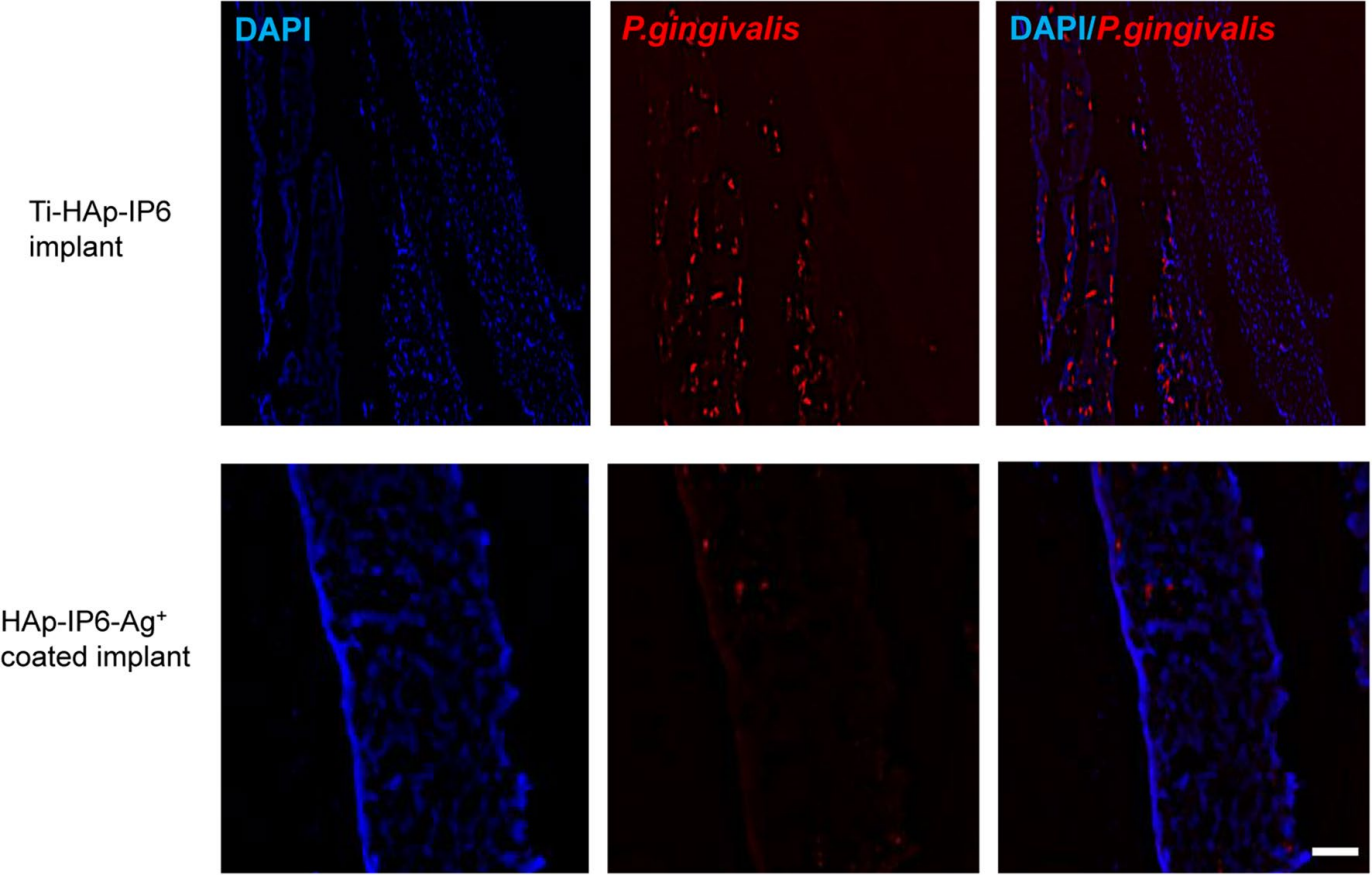
*Porphyromonas gingivalis* infection is significantly antagonized by Ag-coated implants. 4.37 × 10^8^ CFU/ml *P. gingivalis* were transplanted into femoral bone marrow cavities of eight-week-old wild-type mice at the same time that titanium rods were surgically implanted. After 14 days, mice were euthanized, and rods were removed to prepare sections for analysis with mouse anti*-P. gingivalis* mAb followed by Alexa546-conjugated goat anti-Mouse IgG. Nuclei were stained with DAPI. Sections were observed under a fluorescence microscope. Bar = 100 μm. Representative data of at least two independent experiments are shown.

We confirmed these findings in immunohistochemical analysis and, using an anti-*P. gingivalis* antibody, were able to detect *P. gingivalis* in bone marrow cavities, indicating that infectious osteomyelitis by *P. gingivalis* was established in bone marrow cavities implantated with HAp-IP6-Ti at day 14 (Fig. 4). However, such *P. gingivalis* infection in bone marrow cavities was clearly eliminated by HAp-IP6-Ag^+^–Ti rod implantation (Fig. 4).

**Figure 5:**
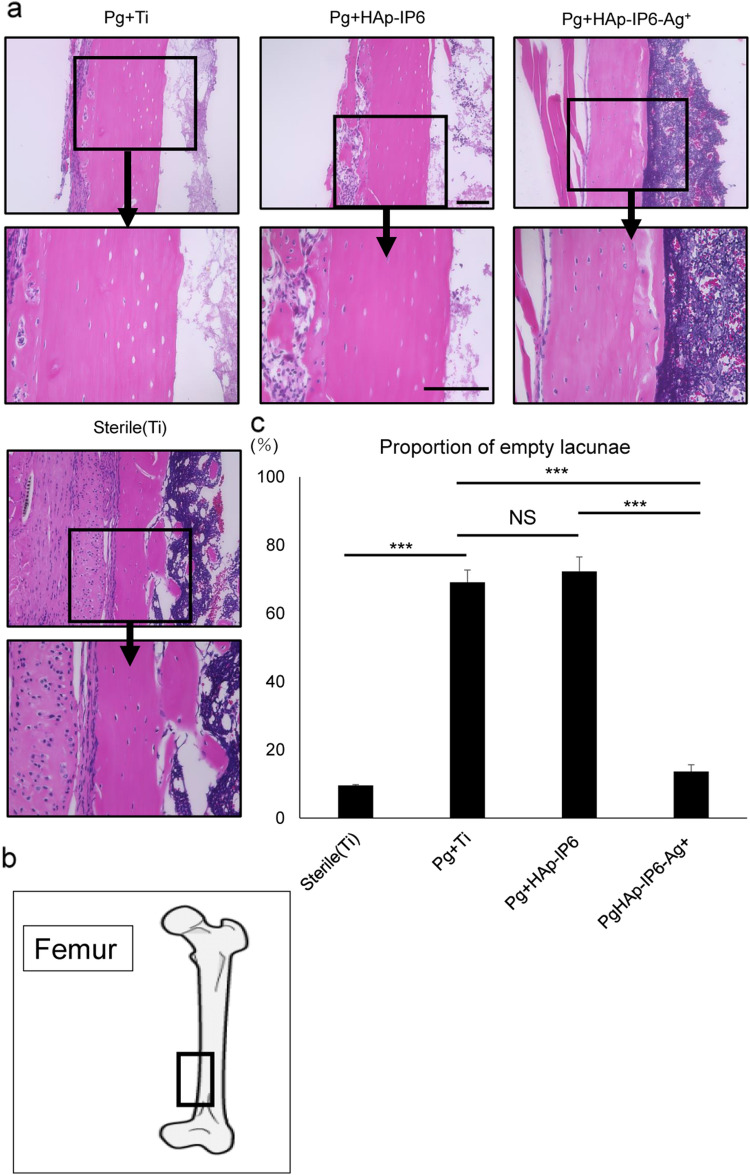
Histological evaluation of control and silver ion implant groups. (**a** and **b**) 4.37 × 10^8^ CFU/ml *Porphyromonas gingivalis* were transplanted into femoral bone marrow cavities of eight-week-old wild-type mice simultaneously with surgical implantation of titanium rods. Rods implanted without *Porphyromonas gingivalis* (sterile + Ti) served as sterile controls. After 14 days, mice were euthanized and rods were removed to prepare sections for hematoxylin and eosin staining at the site highlighted in (**b**). Lower images represent magnifications of squared regions in upper boxes. (**c**) Quantification of analysis shown in (**a**). Shown is the mean relative percentage of empty lacunae among total lacunae ± SD (***p < 0.001; by the Mann–Whitney test). Scale bars in b = 100 (upper) or 20 (lower) μm. Representative data are shown of at least two independent and identical experiments each with n = 5.

**Figure 6:**
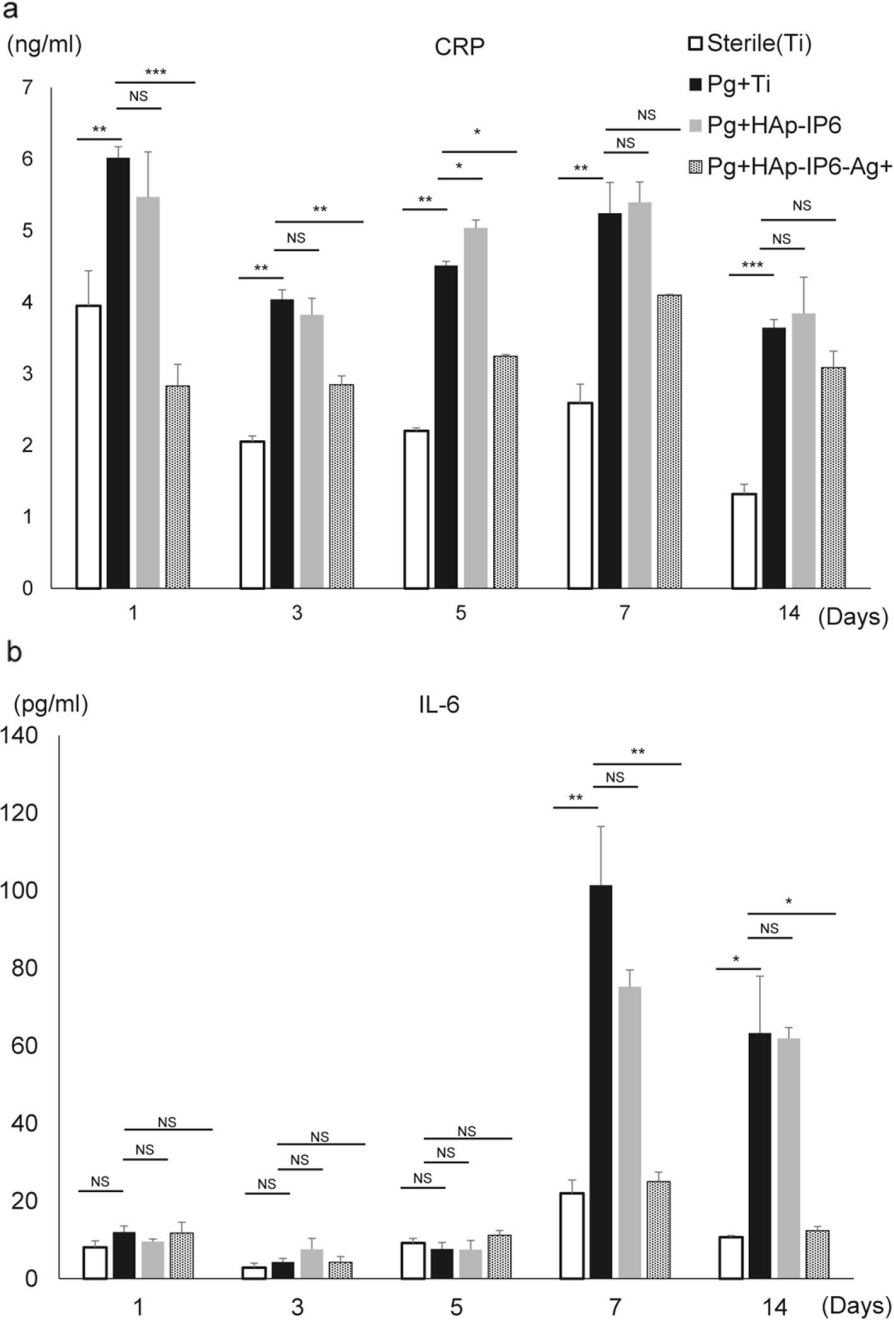
Serological evaluation of control and silver ion-coated implant groups. (**a** and **b**) 4.37 × 10^8^ CFU/ml *Porphyromonas gingivalis* were transplanted into bone marrow cavities of femoral bones of eight-week-old wild-type mice simultaneous with surgical placement of test (HAp-IP6-Ag^+^) and control (HAp-IP6) titanium rods. Rods implanted without *Porphyromonas gingivalis* (sterile + Ti) served as sterile controls. Peripheral blood was collected from mice on indicated days after surgery and serum CRP and IL-6 levels were measured by ELISA. Mean serum CRP (**a**) or IL-6 (**b**) levels ± SD at indicated time points are shown. (n = 5, ***P < 0.001; NS, not significant; by the Mann–Whitney test). Representative data of at least two independent experiments are shown.

We then used histology and biomarker analysis to evaluate effects of Ag^+^ coating of rod implants in mice (Figs. 5, 6). To do so, we established infectious osteomyelitis models of *P. gingivalis’s* with HAp-IP6-Ag^+^–Ti or HAp-IP6-Ti rods as above, collected peripheral blood samples on days 1, 3, 5, 7 and 14, and then euthanized animals for histological analysis of the femur on day 14. Sterile controls were included as the Sham group. Examination of femoral bone revealed that the extent of infectious osteonecrosis, based on formation of empty lacunae, was significantly less in HAp-IP6-Ag^+^–Ti compared to control mice (Fig. 5a–c). Relevant to biomarkers, levels of C-reactive protein (CRP), an early inflammatory marker used clinically to monitor infection and inflammation, were significantly lower in blood of HAp-IP6-Ag^+^–Ti mice at early stages after surgery (Fig. 6a). Likewise, serum levels of the inflammatory cytokine IL-6 were significantly lower in HAp-IP6-Ag^+^–Ti relative to control mice by seven days after surgery (Fig. 6b).

## Discussion

To maintain patients’ ADL and QOL, methods to prevent implant-associated infection after bone implant are crucial, as these infections are difficult to treat and if severe can require removal of the implanted device to eradicate infection^24^. Various methods are used to block or minimize implant-associated infection, such as air purification via use of HEPA filters or antibiotic treatment at the time of surgery^10,25,26^. Nonetheless, these approaches are partially successful, and implant-associated infections still occur^27^. Here, we demonstrate that coating a titanium implant with silver ions can prevent implant-associated infections, even those induced by anaerobic bacteria.

To date, strategies to modify implant surfaces have been considered to prevent implant-associated infection, such as treatment with antibiotics, iodine or silver coating^28–31^. Coating with antibiotics is effective only against some bacteria, and minimally effective against multi-drug resistant bacteria such as methicillin-resistant *Staphylococcus aureus* (MRSA). Although the full spectrum of silver’s antibacterial activity has not yet been defined, silver ions are reportedly effective against the aerobic bacteria *Staphylococcus aureus (S. Aureus)*^14^. Here, we show that silver coating is also effective against anaerobic bacteria infection in vitro and in vivo, even in the bone marrow cavity. This latter finding is particularly significant, as oxygen has been thought critical for antibacterial activities of silver^32^.

*P. gingivalis* infection is typically seen in oral or maxillofacial bones; however, creating infectious osteomyelitis models in mouse via implantation in those bones is technically difficult due to animal size. Instead, we were successful in establishing infectious osteomyelitis in mice by *P. gingivalis* infection with implanting titanium rods in femoral bone marrow cavities. That analysis supported the idea that silver coating of implants provides antimicrobial activities against *P. gingivalis* in those cavities.

*P. gingivalis* is known to form biofilms on implants^33^. Thus, the antimicrobial activity of an ionic silver coating is likely due to inhibition of biofilm formation. Indeed, coating of implants with ionic silver has been shown to inhibit biofilm formation by *S. Aureus*^34^. However, *P. gingivalis* is reportedly a late colonizer during biofilm formation and adheres to biofilms previously formed by primary colonizers such as *Streptococcus Gordonii*^35^. In our animal models, we transplanted *P. gingivalis* only, without primary colonizers, and did not determine whether an ionic silver coating would inhibit biofilm formation by *P. gingivalis*. Further studies are needed to investigate additional effects of ionic silver coating on the complex process of biofilm formation. More complex imaging and biofilm studies are a future direction of the effects of ionic silver coating on the inhibition of biofilm formation implants.

Others have reported that exposing cells to a metal material coated with silver ions using a combination of HAp and IP6 is not toxic to cells^14^. Indeed, we found that *P. gingivalis*-dependent osteonecrosis was significantly decreased in the presence of silver ion-coated rods made of titanium, the material most frequently used in bone implants. Some report that a high temperature is required to silver-coat implants^36,37^. Here, we show that titanium rods can be coated with ionic silver using HAp and IP6 without high temperature, and that those rods exhibit anti-*P. gingivalis* activity. This suggests that comparable methods could be applied to coat non-metallic implant material, such as plastic or polyethylene, with silver ions without using high temperatures. Thus, overall our study suggests that antimicrobial implants against a wide range of bacteria could be made from a variety of materials.

## Materials and methods

### Antibacterial effect of Ag^+^ ions and ionic-silver coating

*Porphyromonas gingivalis* (*P. gingivalis*) W83 was grown anaerobically (5% CO2, 10% H2, balanced N2) in BHIHM medium, which consisted of BactoTM Brain Heart Infusion (BHI; Becton Dickinson, Franklin Lakes, NJ, USA), 7.7 μM hemin, and 2.9 μM menadione. An aliquot (50 μl) of a late-log *PG* culture (~ 0.3 optical density 650 nm (OD650)) was streaked uniformly across a BHIHM agar plate using a disposable inoculation loop. BHIHM agar consisted of 1.5% (w/v) BactoTM Agar (Becton Dickinson) in BHIHM medium. Then, unmodified (control) Ti, HAp-Ti, HAp-IP6-Ti, or HAp-IP6-Ag + (5)–Ti pins were separately placed on the agar and incubated 5 days anaerobically at 37 °C. The inhibition zone was calculated using equation, where D1 and D2 are the area of the respective inhibition zone and the testing pin: Inhibition zone = D1–D2 (Fig. 2). We also spotted twofold serial dilutions of AgNO_3_ (2 μl each) on the agar and incubated plates 48 h anaerobically at 37 °C. *P. gingivalis* growth was determined by measuring the OD650 with a SpectraMax Plus 384 (Molecular Devices, Sunnyvale, CA, USA).

#### Mouse infectious osteomyelitis model

C57BL/6 background wild-type mice were purchased from Sankyo Labo Service (Tokyo, Japan). Mice were maintained under specific pathogen-free (SPF) conditions in animal facilities certified by the Keio University Institutional Animal Care and Use Committee, and animal protocols were approved by that committee. Mice were housed up to 5 per cage and kept on a 12 h light/dark cycle. Sterile distilled water and a standard diet (CLEA Rodent Diet CE-2, Japan) were available ad libitum*.* All mouse studies were performed in accordance with Institutional Guidelines on Animal Experimentation at Keio University of The Keio University Institutional Animal Care and Use Committee. For surgery, all mice received a mixture of ketamine (100 mg/ kg) and xylazine (10 mg/kg) by intraperitoneal injection as anesthesia, and the skin of the left knee was sterilized with povidone iodine, as previously described^38,39^. A skin incision was made over that knee, and the distal femur was exposed. A drill and 23G needle were used to make a hole at the distal end of the femur, and a 0.5 × 8-mm titanium alloy bar was inserted into the mouse femur along with an inoculation of *P. gingivalis* 4.37 × 10^8^ CFU/ml) (for the HAp-IP6-Ag^+^ coating implant group, N = 6). The same technique was used for the control Ti-HAp-IP6 implant group (N = 6). Mice were monitored for a week. This study is reported in accordance with ARRIVE guidelines.

#### Detection of *Porphyromonas gingivalis* by a fluorescence probe in vivo

To visualize an infectious region by *P. gingivalis* osteomyelitis, we used the IVISense Bacterial 750 Fluorescent Probe (Chemiluminescent reagent: Summit Pharmaceuticals International Co.). This system detects and monitors the extent of bacterial growth in animals based on fluorescent intensity. Specifically, we simultaneously inoculated the femur of test mice with *P. gingivalis* and intraperitoneally administered 100 μl of the NIR-fluorescent bacterial detection probe. Mice were anesthetized and monitored at days 1, 3 or 7 after surgery by inhalation of aerosolized 1.5% isoflurane mixed with oxygen. The NIR probe was injected on each imaging day (PODs 1, 3, and 7), and the bacterial probe fluorescence was captured using the trans-illumination feature of the IVIS® Lumina optical-imaging system.

#### Histological and fluorescent immunohistochemical analyses

We collected femur specimens on day 14 after surgery for histological analysis. To do so, animals were euthanized, femurs removed and separated from soft tissues, and samples were then fixed in 4% PFA in 0.1 M PBS, demineralized with 10% ethylenediaminetetraacetic acid, embedded in paraffin, cut into 5-μm-thick sections, and Hematoxylin and Eosin-stained (HE). Empty lacunae were detected in femur sections following HE staining, and the percentage of empty lacunae was calculated relative to total (empty + undamaged) lacunae.

For immunohistochemistry, sections were subjected to microwave treatment for 10 min in 10 mM citrate buffer solution (pH 6.0) for antigen retrieval, as described^40^. After blocking 1 h with 3% BSA in PBS, sections were stained using mouse anti*-P. gingivalis* mAb (D376-3 1:100 Medical & Biological Laboratories Co.,LTD, Tokyo, Japan), followed by Alexa546-conjugated goat anti-Mouse IgG (#A-11030 1:200; Invitrogen, Carlsbad, CA, USA). Nuclei were visualized by DAPI (#D1306 1:750; Wako Pure Chemicals Industries, Osaka, Japan).

### Cell cytotoxicity assay.

#### Cell culture

Murine osteoblastic cells (MC3T3-E1) were cultured in α-MEM (Sigma-Aldrich, St. Louis, MO) containing 10% heat-inactivated fetal bovine serum (FBS) (SAFC Biosciences) and GlutaMax (Invitrogen, Carlsbad, CA) at 37 °C under 5% CO_2_ air. Medium was changed every 48 h, and cells were passaged using TrypLE™ Express Enzyme (1X) with no phenol red (Thermo Fisher Scientific K.K, Massachusetts, USA) once a week when they reached 90% confluence. Proliferation of MC3T3-E1 cells cocultured with HAp-IP6-Ag + -coated or uncoated Ti-HAp-IP6 implants was examined using 12-well Multiwell TC Plates (Corning, NY). First, MC3T3-E1 cells were seeded onto plates at a density of 6 × 10^4^ cells per well in 2 ml medium and incubated 24 h. Then, an HAp-IP6-Ag + -coated or uncoated Ti-HAp-IP6 implant was placed in each well. Cells cultured for 3 days and observed under a phase-contrast microscope. Finally, cells were harvested and counted.

#### Enzyme-linked immunosorbent assay (ELISA)

Serum CRP and IL-6 ELISA assays were undertaken following the manufacturer's instructions (R&D Systems, Minneapolis, MN, USA) using a multiple plate analyzer (Cytation 5, BioTek Instruments, Inc., Winooski, VT, USA).

#### Statistical analysis

Quantified data are shown as means ± SD. Statistical significance of differences between or among groups was evaluated using Student’s t test, the Mann–Whitney *U* test or a one-way analysis of variance (ANOVA), respectively, using statistical software (version 25; SPSS Inc., Chicago, IL, USA) (*P < 0.05; **P < 0.01; ***P < 0.001; NS, not significant, throughout the paper) as previously described^40^.

## Supplementary Information

Supplementary Figure S1.

## Data Availability

The datasets used and/or analysed during the current study available from the corresponding author on reasonable request.
