# Supplementary material for: An ionic silver coating prevents implant-associated infection by anaerobic bacteria in vitro and in vivo in mice
Source: Sci Rep. 2022 Nov 1;12:18387. doi: 10.1038/s41598-022-23322-6 (PMC9626628; doi:10.1038/s41598-022-23322-6)
Supplement: Supplementary file 1 — Supplementary Figure S1. [file 41598_2022_23322_MOESM1_ESM.pdf]

An ionic silver coating prevents implant-associated infection by anaerobic bacteria *in vitro* and *in vivo* in mice

Tomoya Soma[1], Ryotaro Iwasaki[1], Yuiko Sato[2,3], Tami Kobayashi[2,4], Eri Ito[5], Tatsuaki Matsumoto[2], Atsushi Kimura[2], Fuka Homma[1], Keitarou Saiki[6], Yukihiro Takahashi[6], Kana Miyamoto[7], Morio Matsumoto[2], Masaya Nakamura[2], Mayu Morita[1], Ken Ishii[2,8], Seiji Asoda[1], Hiromasa Kawana[1, 9], Zhu Xingyu[10], Mamoru Aizawa[10], Taneaki Nakagawa[1] and Takeshi Miyamoto[2, 3, 4, 7]

[1]Division of Oral and Maxillofacial surgery, Department of Dentistry and Oral Surgery, Keio University School of Medicine, 35 Shinano-machi, Shinjuku-ku, Tokyo 160-8582, Japan, [2]Department of Orthopedic Surgery, Keio University School of Medicine, 35 Shinano-machi, Shinjuku-ku, Tokyo 160-8582, Japan, [3]Department of Advanced Therapy for Musculoskeletal Disorders II, Keio University School of Medicine, 35 Shinano-machi, Shinjuku-ku, Tokyo 160-8582, Japan, [4]Department of Musculoskeletal Reconstruction and Regeneration Surgery, Keio University School of Medicine, 35 Shinano-machi, Shinjuku-ku, Tokyo 160-8582, Japan, [5]Institute for Integrated Sports Medicine, Keio University School of Medicine, 35 Shinano-machi, Shinjuku-ku, Tokyo 160-8582, Japan, [6]Department of Microbiology, The Nippon Dental University School of Life Dentistry at Tokyo , 1-9-20 Fujimi, Chiyoda-ku, Tokyo 102-8159, Japan, [7]Department of Orthopedic Surgery, Kumamoto University, 1-1- Honjo, Chuo-ku, Kumamoto 860-8556, Japan, [8]Department of Orthopaedic Surgery, School of Medicine, International University of Health and Welfare (IUHW), 852 Hatakeda, Narita City, Chiba, 286-8520, Japan, [9]Department of Oral and Maxillofacial Implantology, School of Dentistry, Kanagawa Dental University, 82 Inaoka-cho, Yokosuka, Kanagawa, 238-8580, Japan, [10]Department of Applied Chemistry, School of Science and Technology, Meiji University, 1-1-1 Higashimita, Tama-ku, Kawasaki, Kanagawa, 214-8571, Japan.

Running title: *Silver ions antagonize anaerobic bacterial infection*

Correspondence should be addressed to: T. M., Department of Orthopedic Surgery, Keio University School of Medicine, 35 Shinano-machi, Shinjuku-ku, Tokyo 160-8582, Japan or Department of Orthopedic Surgery, Kumamoto University, 1-1- Honjo, Chuo-ku, Kumamoto 860-8556, Japan. TEL: 81-3-5363-3812, FAX: 81-3-3353-6597, e-mail: miyamoto@z5.keio.jp or miyamoto.takeshi@kuh.kumamoto-u.ac.jp

Supplementary Figure Legends

Figure S1. No fluorescence signals were detected in sterile controls.

Titanium rods were implanted into femoral bone marrow cavities in eight-week old wild-type mice without *P.gingivalis*. Then, mice were intraperitoneally injected with a NIR-fluorescent bacterial detection probe, and the bacterial probe fluorescence was captured using the trans-illumination feature of the IVIS® Lumina optical-imaging system. No fluorescence signals were detected 1 day after surgery in the sterile controls (Ti rod without *P.gingivalis* transplantation).

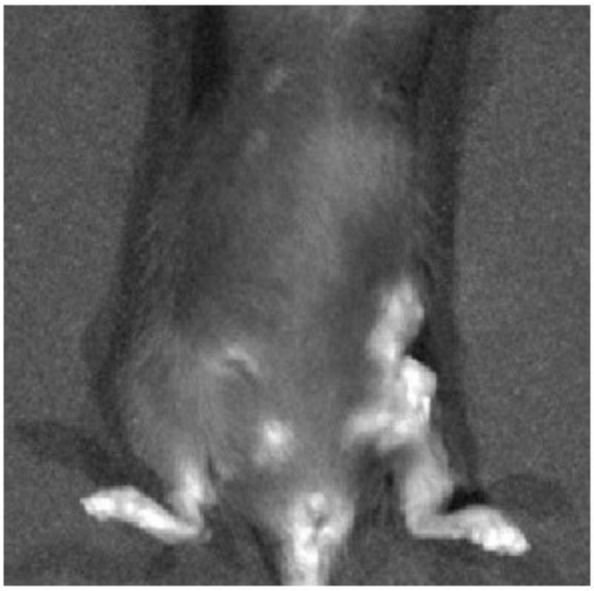

Figure S1 Soma T et al
